# Support for a Weight‐Inclusive Curriculum? Exploring Partner Perspectives and Influences on Nutrition Education in Vermont High Schools

**DOI:** 10.1111/josh.13510

**Published:** 2024-10-24

**Authors:** Deborah Hinchey, Bernice Raveche Garnett, Janet Gamble, Lizzy Pope

**Affiliations:** ^1^ Public Health Sciences University of Vermont Burlington VT; ^2^ Department of Education University of Vermont Burlington VT; ^3^ Food Systems University of Vermont Burlington VT; ^4^ Department of Nutrition and Food Sciences University of Vermont Burlington VT

**Keywords:** weight‐normative, weight‐inclusive, high school nutrition education

## Abstract

**Background:**

The weight‐normative approach to nutrition education dominates health education programming across the United States, despite evidence that this paradigm contributes to negative outcomes including weight cycling, bias and stigma, the development of disordered eating behaviors, and weight‐based bullying.

**Methods:**

This study investigates perspectives of 10 potential partners with interest in and potential to influence nutrition education. Through qualitative interviews and document analysis, researchers explored support for a weight‐inclusive curriculum and factors that influence high school nutrition curricular content and implementation.

**Results:**

Findings indicate that partners hold both weight‐inclusive and weight‐normative values, guidance around nutrition curricular content is lacking, and state‐level policy is crucial to the valuing and implementation of consistent curricula.

**Implications for School Health Policy, Practice, and Equity:**

Lack of guidance or mandated curricular content contributes to inequities across the state. Weight‐inclusive curricular materials are needed. Administration must take an active role in providing access to professional development and state policy support for curricular implementation is essential.

**Conclusions:**

Weight‐inclusive curriculum could serve to improve health outcomes for adolescents. However, successful implementation of, or changes to, health and nutrition curricula will require support and engagement from partners at all ecological levels.

Nutrition education is a crucial component of school health education and has the potential to impact academic success and student health outcomes well beyond the high school years.^1^, ^2^, ^3^ Nutrition education equips students with the knowledge and skills necessary to make health‐promoting decisions around food and supports them in connecting nutritional choices to other aspects of their lives, including the impact of food on emotional and mental health.^1^ However, the majority of kindergarten to 12th‐grade schools across the United States report not teaching about nutrition.^4^

## Weight‐Normative Approaches

The “war on obesity” is regularly a driver for policies, health promotion programs, health care systems, and educational interventions across the United States.^4^, ^5^, ^6^ This “war” is consistent with a weight‐normative approach to health, one that posits that weight is a primary determinant of health, that it is an individual‐level problem to be managed, that food has moral value, and that weight loss improves health outcomes.^5^, ^6^ The obesity and weight‐focused dominant narrative in the United States and its implied solution of weight loss fails to recognize underlying determinants of body weight, mischaracterizes the connection between body weight and health, and is a profoundly ineffective and harmful approach to improving health outcomes,^7^ contributing to weight cycling, weight bias, and weight‐based bullying.^4^, ^5^, ^6^ Weight‐normative approaches support the notion that there is an ideal body weight that is achievable for all individuals, leading to increased body shame, internalized stigma, body dissatisfaction, and eating disorders.^6^ Weight stigma and anti‐fat bias are also contributing factors in the development of eating disorders in adolescents.^5^, ^8^ This is especially significant given the rise in eating disorders in adolescents during the Covid‐19 pandemic.^9^, ^10^

## Weight‐Inclusive Approaches

In contrast to weight normativity, weight‐inclusive approaches argue that body size does not equate to health^4^, ^5^, ^6^ and that it is health behaviors, such as exercising, eating a healthy diet, not smoking, and limiting alcohol consumption, that are the underlying contributors to health and longevity, rather than weight.^5^, ^6^ The weight‐inclusive approach recognizes the Social Determinants of Health (SDOH): that the environments in which people live shape what food, transportation, and recreational opportunities individuals can access.^11^, ^12^, ^13^ This approach has myriad benefits including improved self‐care practices, an increase in health‐promoting behaviors, improvements in physiological measures like blood pressure and blood lipids, and improvements in mental health outcomes such as eating disorder development.^4^

## Partners and Influences

**Figure 1:**
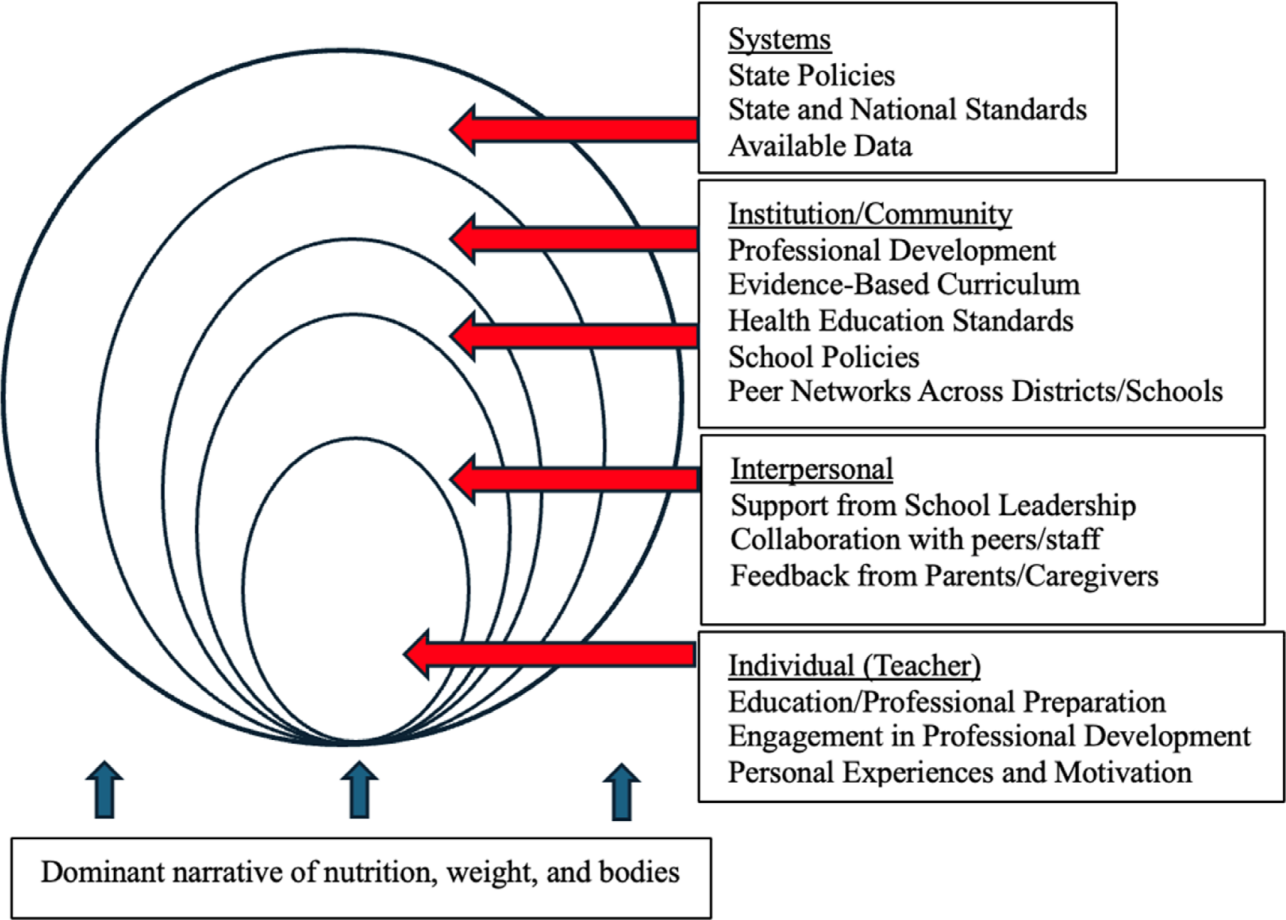
Multiple Levels of Influence on Curricular Decisions

The education system, as a space in which dominant beliefs around health are reinforced and internalized^14^ is in a unique position to shape the narrative around nutrition, weight, and bodies. Educators support the inclusion of eating disorder and weight‐based bullying prevention in health curriculum,^15^ and implementing comprehensive weight‐inclusive curriculum offers an opportunity for primary prevention to this end. The literature provides ample evidence that health and nutrition curriculum content and implementation are influenced by individuals and forces at multiple levels beyond teacher decision‐making, including peers/colleagues, school administration, available curriculum, and professional development opportunities, and state policies (see Figure 1). Given the potential for harm caused by the weight‐normative, obesity‐focused approach in the United States, it is imperative that schools offer a counter‐narrative.

Successful implementation of or changes to health and physical activity curriculum require engagement from cross‐school partners.^16^ Schools that have established health councils made up of varied partners, and schools that facilitate collaboration across units experience greater support for health education overall.^16^, ^17^, ^18^ Strong collaborations between food service staff, health education teachers, school nurses, and other health‐related staff facilitate consistent messaging around nutrition and an improvement in the overall school food environment.^19^ School administrator support is crucial to successful implementation of health and physical education programming, as administrators can impact entire school and community culture. Schools in which administrators demonstrate strong support for and engagement with health program implementation are more likely to achieve intended program outcomes.^16^, ^20^, ^21^ School administrators also plays a crucial role in supporting teachers in attaining needed professional development.^22^

Access to sustained and consistent professional development, rather than short‐term/single‐day workshops,^18^ in specific content areas including nutrition, improves teachers' ability to deliver curriculum.^18^, ^19^ Professional development is an effective way to enhance health teachers' sense of preparedness, subject‐specific content knowledge, and pedagogical approaches to the implementation of critical health literacy lessons.^18^ Additionally, being provided with curriculum to adapt to their classrooms is an effective tool to increase a teacher's confidence in implementing a nutrition education program.^18^

Policies at multiple levels can have a significant positive impact on the implementation of and consistency in delivering health education.^22^, ^23^, ^24^ When exploring teacher attitudes toward sexual health education in Minneapolis, Eisenberg et al.^23^ found that teachers felt strongly that policies at the federal level exert significant influence on the culture around sexuality education and that state‐level law mandating required curricular content for comprehensive sexual health education would make implementation easier and ease pressure on administrators. Support from school administrators, school districts, and states to implement policy changes is key to the success of health education programs.^22^, ^24^

The purpose of this study was to explore the perspectives of potential partners that have influence on and interest in nutrition education curricular development and implementation in Vermont high schools. Researchers endeavored to answer the following questions:

1. What are the unique perspectives of potential partners at various ecological levels regarding nutrition education in Vermont?
2. What multi‐level factors influence Vermont high school curricular content related to nutrition, weight, and bodies?

## METHODS

For this study, an exploratory, single case‐study design was used, and a common case was identified.^25^ Researchers defined the case as nutrition education in Vermont high schools; nutrition education in Vermont exists as a complex system, with multiple interconnected elements that influence classroom curriculum. This paper specifically explores the perspectives of key partners that have an interest in and influence over the content and delivery of health and nutrition education in schools. This research is nested within a larger, mixed‐methods project; one component of the larger project is designing and implementing a weight‐inclusive high school nutrition curriculum and offering professional development to teachers. It is essential to understand the perspectives of partners with influence over the adoption of a new, weight‐inclusive curriculum once it is developed.

### Participants

Researchers conducted 10, qualitative, semi‐structured interviews with partners in Vermont high school nutrition education. Partners were identified by their potential serve as champions for a new curriculum—to influence content or adoption. According to Shea et al.^26^ (p. 1), a champion is an “individual who works within an organization and who dedicates themselves to promoting a change within the organization, such as implementing a new intervention or a new quality improvement effort.” Inclusion criteria comprised the following:

1. High interest in nutrition curriculum
2. Holding a role at the interpersonal, institutional, community, or policy levels with the potential for high influence on nutrition curriculum adoption at the high school level

**Figure 2:**
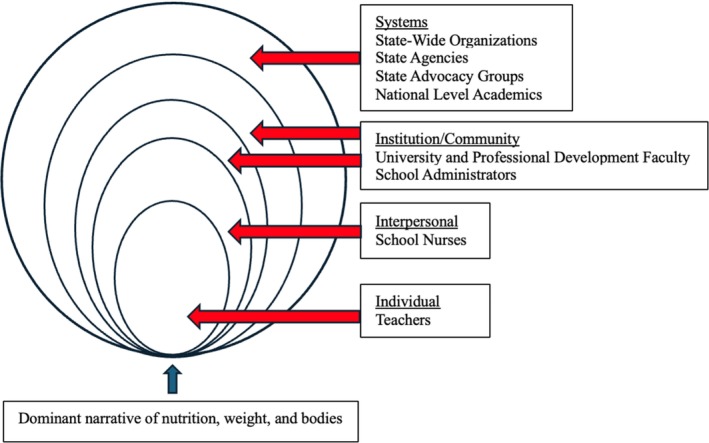
Partners at Multiple Levels in Vermont

Partner identification was informed by the literature and through purposeful and theoretically driven sampling. Using the levels of the social‐ecological model (SEM) as the theoretical framework, individuals and their associated institutions were selected to ensure information‐rich participants at each of the SEM levels, beyond the individual.^27^ Because of the depth of connections between the research team on this project and the school health and nutrition professional networks, multiple discussions within the team yielded a significant list of potential partners. Potential partners were analyzed based on their professional positions as related to the levels of the SEM, their level of interest in nutrition education and their potential to exert influence over the content or adoption of nutrition curriculum (see Figure 2).

The most challenging to assess was the potential for influence over curricular adoption. Informed by Shea,^26^ Balane et al,^28^ and Varvasovszky and Brugha,^29^ influence was defined by the following, (1) whether the partner has direct influence over individuals, institutions or policy processes that would translate into mandates or requirements for curricular content or adoption, (2) whether the partner has the ability to influence individuals or institutions in the form of enforcement of policies, recommendations for practice and/or curricular content inclusion (3) whether the partner has the potential to shape and influence thoughts or opinions around nutrition curriculum in high schools. All partners identified have high on and interest in nutrition curriculum in high schools in Vermont.

### Instrumentation

The team of researchers is comprised of interdisciplinary expertise including public health, nutrition, school health, community schools and eating disorder prevention and treatment. The team developed interview questions to explore the unique perspectives of individuals and their associated organizations regarding nutrition education in high schools. Interviews lasted approximately 30 minutes and took place via Microsoft Teams. The interviews occurred during January–March 2023 and followed a semi‐structured format to allow partners to expand on the topics being discussed. The websites associated with partner organizations were also reviewed, along with any documents related to health and nutrition education including policies, available curriculum, and professional development opportunities.

### Data Analysis

As described in Creswell and Poth,^27^ and Saldana,^30^ the researchers took a lean coding approach to the data and began with a‐priori (provisional) codes informed by the literature review, theoretical framework, and research questions. A draft codebook was shared with the research team and each member of the team coded the first transcript to ensure consistent interpretation and application of codes. The team then met to discuss application of codes, emergent codes, and potential updates to the codebook. Many of the initial codes were too broad and the team was able to suggest expanded and more specific codes to better address the research questions. Based on discussion and feedback the codebook was expanded,^31^ refined, and finalized. Next, researchers initiated second cycle coding, studying the frequency with which codes were applied, and wrote memos reflecting on themes that were emerging.^25^, ^30^ Finally, pattern coding was applied, grouping coded excerpts into themes.^30^ An essential element of the coding process was to analyze responses through the lens of the theoretical framework while applying a critical lens.

A review of additional sources of evidence was an important addition to the data collection and analysis for this study. District, school, and organizational websites associated with partners were reviewed by the research team, as well as the websites for state‐wide professional organizations and professional development agencies. Consistent with case study methodology, researchers analyzed documents with the purpose of triangulation: corroborating and deepening understanding of findings from partner interviews through multiple measures of the same phenomena.^25^, ^32^ The codes used in the analysis of partner interviews formed the foundation for document analysis.

## RESULTS

Three themes were generated through analysis of the interview data, (a) partners in positions with high influence hold both weight normative and weight inclusive perspectives on nutrition education, (b) guidance around nutrition curriculum content is needed and essential, and (c) state‐level policy is crucial to the valuing and implementation of consistent nutrition curriculum.

### Partners in Positions With High Influence Hold Both Weight Normative and Weight Inclusive Perspectives on Nutrition Education

Nine of the partners interviewed expressed weight‐inclusive values, including body‐size acceptance, health at every size and anti‐diet principles. Partner #7 shared: “We know that the biggest risk factor for people right now is weight cycling… I think intuitive eating is probably one of the greatest things that we've introduced to our people.” Weight cycling is often cited as an outcome of a weight‐based approach to health, and intuitive eating is an example of a weight‐inclusive approach.^6^ Eight of the partners also expressed weight‐normative values, such as advocacy for the inclusion of the body mass index in the curriculum, and support for the weight‐health connection and the obesity‐as‐disease paradigm. In many of the interviews, both weight‐normative and weight‐inclusive values were expressed within the same excerpts, as shown in the example below. The participant affirms the obesity as a disease approach and the weight‐health connection, while also articulating weight‐inclusive principles such health at every size and the impact of the SDOH:

I don't like the idea of focusing on obesity and weight and body size all the time, and especially focusing on it as something that individuals are meant to change. Because we know that individuals have very limited power to do that, and that it just makes things worse. Right? And does result in picking up disordered eating and stigmatization. But then I'm also not on board with, I think, with the other side of them of just being like, “Oh, well, this is all junk science. Obesity doesn't exist.” Because we know that it sure does. And it is something that is associated with worse health outcomes, and it's not something that's great for people's bodies over time. Partner #5

The expression of weight‐inclusive and weight‐normative values together was clearly articulated when partners shared their perspectives on nutrition/weight‐related content areas that are important for high school students to learn about in health class, exemplified in the following quote discussing framing exercise in ways that both support health but also as a path toward weight loss:

I think they just need to tie the concepts together to: what is it that I want to do? Do I want to run a marathon? Do I wanna just do what I have to do to stay fit because I don't really like physical activity. And weight management and where's a healthy zone? What are the risk factors for not being at a healthy weight, or at least in a healthy zone? And how does that play out? Because I think a lot of people find out too late that the extra weight they're carrying ruined their knees or their hips or their feet. Partner #4

Two of the partners did discuss that there are efforts in their own organizations to shift the narrative from one focused on weight normativity toward weight inclusivity. For example:

We're in that learning phase about who's talking about this, what does any science say about if we were gonna not focus on scales… is there enough science to say that somebody who is clinically obese is absolutely headed for really bad outcomes. I don't think there is, but I don't think there's enough science the other way yet either. Of course, as you know we need to really be leaning back on the science stuff. So, we're trying to navigate what we do know in science, what we think is right, and then sort of figure out where we want to go from there. Partner #10

It is interesting to note that only two of the partners referenced the multiple levels of influence on nutritional choices faced by high school students, beyond the individual level. Partner #3 said: “How to, you know, help them learn about anything that's valuable to them when they have no say over what is in their cupboards, if there's anything in their cupboards at all.”

### Guidance Around Nutrition Curriculum Content Is Needed and Essential

Eight partners discussed a need for support and guidance in the teaching of health and nutrition through professional development for health teachers, a shared curriculum and policy‐level intervention to ensure consistency. Partner #3 shared that “there's not a lot of resources to support it in terms of lesson plans… There's no directed PD at health teachers like there is you know for maybe math teachers and literacy teachers.” While Partner #6 said:

We need good content. If you can build great content for us to draw from, that would be great. I think schools are going to have health class, but what happens in that health class, we could probably use help with.

All of the partners interviewed commented on the value of health and nutrition education to the high school curriculum, demonstrated in the following quote:

If we don't teach it in high school, and give them the knowledge, the information, but more importantly the skills to access information to understand information for the future for themselves, or even to help others, that whole concept of health literacy, where else are they gonna get it? Partner #1

### State‐Level Policy Is Crucial to the Valuing and Implementation of Consistent Nutrition Curriculum

Partners noted that, while Vermont does require that health and nutrition education be a component of the high school curriculum, there is inherent inequity in only mandating, broadly, that health be taught but not providing specifics to guide content:

There is a clear role that is defined by our education quality standards by statute. So, the schools do have a role, a very important role in carrying out comprehensive health education. However, it is very inconsistent and inequitable across our school districts. So, some take it on and do a really great job, and then others don't even teach health. Partner #4

Partners also identified a desire for consistent curriculum across the state:

I think it would be awesome if we had a nutrition program that was consistent across the state. Like, I say this all the time. Health is consistent. It doesn't matter where you are, or what district you're in, it's the same. It's the same content, it's the same everything. But yet we have this thought process in Vermont where every district needs to have their own locus of control, which changes the dialogue about what we're teaching, and how we're teaching it. But health is health, is health, right? Partner #9

Partners also expressed a shared desire for health education broadly to be a more valued component of the high school curricular landscape. Partner #2 said: “Gosh, if we improved our health education, maybe it would help us to address some of these other challenges that our kids are facing.”

## DISCUSSION

Collaboration with peers,^19^ support from principals,^16^, ^20^ state‐level policies,^22^, ^23^, ^24^ professional development and curricular materials^18^ are all essential to successful health and nutrition education at the high school level. As such, for this study, current literature informed the identification of partners, including school nurses, principals, individuals, and institutions that inform curricular content (professional development instructors and teacher‐trainers) and entities that drive policies (state‐level institutions and government). It was very clear from the interviews that partners recognize the crucial role that nutrition education plays in the overall health education curriculum, broadly, and to the health outcomes and academic success of students and are supportive of nutrition education being included and valued at the high school level.

It is essential to note that the partners interviewed expressed both weight‐inclusive and weight‐normative values regarding nutrition, weight, and bodies. As is made clear in the literature, weight‐normative approaches to health can lead to increased body shame, internalized stigma, body dissatisfaction, and eating disorders.^5^, ^6^, ^8^ This is especially significant given the rise in eating disorders in adolescents during the pandemic.^9^, ^10^ The narrative of the weight‐normative paradigm supports the notion that being fat is inherently bad,^5^, ^6^ and the rise in anti‐fat messaging throughout the pandemic has been noted as a primary contributor to the rise in disordered eating behaviors.^33^ Weight‐normative perspectives are additionally concerning because of the continued high rates of bullying among high school students, especially those who are labeled as overweight or obese.^8^ It was encouraging to find that all but one of the partners expressed weight‐inclusive values, as a weight‐inclusive approach to high school nutrition education has the potential to support students in developing healthier relationships with food and decrease weight‐based stigma and associated harms.^6^ This, in turn, could improve academic outcomes, as there is an inextricable link between student physical health, mental health, social emotional learning, and academic outcomes.^34^, ^35^

Given the importance of teacher professional development to curriculum content, adoption, and implementation,^18^, ^19^ including teacher confidence,^18^, ^36^, ^37^ identifying that partners feel that there is a dearth of professional development opportunities is instructive. There was a very clear message from partners that there is a need for evidence‐based nutrition curriculum that can be shared with and consistently taught by health teachers across the state and accompanying professional development to support teacher efforts. Supporting teachers identifying current and relevant scientific data, as well as alternative ways of knowing, including personal narratives and gray literature, to inform their teaching around nutrition is essential.^38^ This is a clear gap to be filled in Vermont.

Finally, there was a consensus among the partners that nutrition education plays a key role in the health of high school students. This recognition is consistent with the current literature citing health education, broadly, as key to student academic success and health outcomes beyond the high school years.^1^, ^2^, ^39^, ^40^ Partners expressed concern that health and nutrition education are not valued parts of the high school curriculum in Vermont, citing the state's lack of specificity in content and that schools are not held accountable for health education outcomes. State‐level intervention, according to the partners, is essential for nutrition education curriculum to be implemented consistently and impact student health. This is consistent with the literature that policies at multiple levels impact the implementation of and consistency in delivering health education.^22^, ^23^, ^24^ Furthermore, support from school administrators, school districts, and states to implement policy changes are key to the success of health education programs.^22^, ^24^ Future directions for this research include piloting a weight‐inclusive curriculum with high school teachers across the state of Vermont, and further engagement with partners to facilitate successful development, implementation, and evaluation.

## IMPLICATIONS FOR SCHOOL HEALTH POLICY, PRACTICE, AND EQUITY

The first year of the COVID‐19 pandemic (2020) saw a significant rise in eating disorders in youth.^33^, ^41^, ^42^ Given this, and the high rates of weight‐based bullying in adolescents and young adults, it is crucial that schools begin to implement health education curriculum that is based on weight‐inclusive principles. As partners discussed, the lack of curricular guidance or mandated content contributes to inequities across the state. As one partner noted, while the settings may change, nutritional principles are consistent, and should therefore be consistently taught to all students. There is a need for development of weight‐inclusive nutrition education curriculum materials, and then state policy support for the implementation of these curricula.

School administration must also take an active role in providing the time for and access to professional development. School leadership, and principals in particular, are essential to the prioritization and delivery of health curriculum and play a crucial role in supporting teachers in attaining needed professional development.^22^ Successful implementation of or changes in health and physical activity curricula require engagement from across‐school partners, and schools that facilitate collaboration across units experience greater support for health education overall.^16^ Professional development should therefore not be limited to health education teachers, rather, should engage partners at all levels of the SEM given their potential to impact curricular content and adoption.

### Limitations

Limitations of the data collection for this case study include a small within‐case sample size and limited availability of documents to review. Adding additional partners could have yielded further depth of understanding of the influences on curricular decisions. The research team made significant effort to connect with school board members and members from state government serving on the education committee(s), but requests for interviews were not successful. It is interesting to note that these partners felt that, despite clearly being in roles with the potential to influence curriculum, they did not have any knowledge of health or nutrition education to share. However, despite these limitations, the triangulation of gathered and available data ultimately supported an in‐depth case study.

### Conclusions

If future curricular and professional development interventions are to be successful in impacting nutrition education in high schools, they must be built on a foundation that accounts for various levels of influence, including and perhaps most importantly the perspectives of partners whose interest and positionality afford the power to influence curricular content and adoption. This is important work, as a weight‐inclusive curriculum—a curriculum that challenges dominant societal narratives around body size and health—could serve to not only improve health outcomes and decrease body‐size stigma for adolescents but also have broader societal impact.^43^ Further engagement with the partners that participated in this research could provide needed leverage to mandate and implement a weight‐inclusive curriculum.

### Human Subjects Approval Statement

This study was reviewed by the Chair of the IRB at the University of Vermont using the exempt procedures set forth under 45 CFR 46.104. While the project was exempt from IRB review, researchers followed all human subject protection regulations.

### Conflict of Interest

No conflicts to disclose.
